# Clinical characterization of familial hypercholesterolemia due to an amish founder mutation in Apolipoprotein B

**DOI:** 10.1186/s12872-022-02539-3

**Published:** 2022-03-17

**Authors:** Katie B. Williams, Michael Horst, Millie Young, Christine Pascua, Erik G. Puffenberger, Karlla W. Brigatti, Claudia Gonzaga-Jauregui, Alan R. Shuldiner, Samuel Gidding, Kevin A. Strauss, Devyani Chowdhury

**Affiliations:** 1 Clinic for Special Children, Strasburg, PA USA; 2 Center for Special Children – La Farge Medical Clinic - Vernon Memorial Healthcare, La Farge, WI USA; 3 Penn Medicine Lancaster General Health Data Science & Biostatistics, Lancaster, PA USA; 4 Children’s Hospital of Philadelphia, Philadelphia, PA USA; 5 Regeneron Pharmaceuticals Inc., Tarrytown, NY USA; 6 Division of Cardiology, Nemours Alfred I. duPont Hospital for Children, Wilmington, DE USA; 7 Genomic Medicine Institute, Geisinger Medical Center, Danville, PA USA; 8 Penn Medicine-Lancaster General Hospital, Lancaster, PA USA; 9 Cardiology Care for Children, 1834 Oregon Pike, Lancaster, PA 17601 USA

**Keywords:** Familial hypercholesterolemia, Amish, Apolipoprotein, Cholesterol, Carotid initma-media thickness, Pulse wave velocity

## Abstract

**Background:**

Familial hypercholesterolemia (FH) due to a founder variant in Apolipoprotein B (ApoB^R3500Q^) is reported in 12% of the Pennsylvania Amish community. By studying a cohort of ApoB^R3500Q^ heterozygotes and homozygotes, we aimed to characterize the biochemical and cardiac imaging features in children and young adults with a common genetic background and similar lifestyle.

**Methods:**

We employed advanced lipid profile testing, carotid intima media thickness (CIMT), pulse wave velocity (PWV), and peripheral artery tonometry (PAT) to assess atherosclerosis in a cohort of Amish ApoB^R3500Q^ heterozygotes (n = 13), homozygotes (n = 3), and their unaffected, age-matched siblings (n = 9). ApoB^R3500Q^ homozygotes were not included in statistical comparisons.

**Results:**

LDL cholesterol (LDL-C) was significantly elevated among ApoB^R3500Q^ heterozygotes compared to sibling controls, though several ApoB^R3500Q^ heterozygotes had LDL-C levels in the normal range. LDL particles (LDL-P), small, dense LDL particles, and ApoB were also significantly elevated among subjects with ApoB^R3500Q^. Despite these differences in serum lipids and particles, CIMT and PWV were not significantly different between ApoB^R3500Q^ heterozygotes and controls in age-adjusted analysis.

**Conclusions:**

We provide a detailed description of the serum lipids, atherosclerotic plaque burden, vascular stiffness, and endothelial function among children and young adults with FH due to heterozygous ApoB^R3500Q^. Fasting LDL-C was lower than what is seen with other forms of FH, and even normal in several ApoB^R3500Q^ heterozygotes, emphasizing the importance of cascade genetic testing among related individuals for diagnosis. We found increased number of LDL particles among ApoB^R3500Q^ heterozygotes but an absence of detectable atherosclerosis.

**Supplementary Information:**

The online version contains supplementary material available at 10.1186/s12872-022-02539-3.

## Background

Familial hypercholesterolemia (FH) is a genetic disorder with autosomal dominant inheritance leading to elevated low density lipoprotein cholesterol (LDL-C) levels and predisposition to premature atherosclerosis and cardiovascular disease (CVD) [1]. Symptoms of CVD typically emerge in adults, but atherosclerosis begins during childhood [2–4].

A founder variant in Apolipoprotein B-100 (NC_000002.12; NM_000384.3; *APOB* c.10580G > A; p.Arg3527Gln; previously described as p.Arg3500Gln; referenced here as ApoB^R3500Q^) has a 12% carrier frequency among the Old Order Amish of Lancaster County, Pennsylvania [5]. Adults who are heterozygous for ApoB^R3500Q^ have 58 mg/dL higher LDL-C and are about 4.5 times more likely to have detectable coronary artery calcification [5]. Early identification of the ApoB^R3500Q^ variant in the Amish community may offer an opportunity to prevent or delay development of atherosclerosis by providing treatments to maintain low LDL-C levels [6, 7].

Individuals with FH due to variants in the LDL receptor (*LDLR*) have increased carotid intima media thickness (CIMT) by age 8–10 years, and there may be presence of aortic lesions on magnetic resonance imaging [1]. Coronary artery calcification can be seen in 25% of adolescents with *LDLR* FH [8]. In addition to LDL-C levels, other lipid components, such as Apolipoprotein-B (ApoB), Apolipoprotein A-1 (ApoA-1), LDL and HDL particles (LDL-P and HDL-P), small, dense LDL cholesterol (sdLDL-C), and Lipoprotein(a) [Lp(a)] may also be elevated and associated with atherosclerosis. Less is known regarding atherosclerotic risk in children and young adults with FH caused by variants in *APOB*.

In the current study, we employed both noninvasive cardiac imaging modalities and advanced lipid profile testing to assess premature atherosclerosis risk in a cohort of Amish subjects with ApoB^R3500Q^ and their unaffected, age-matched siblings. By studying a genetically homogenous cohort of subjects with FH and sibling controls, we aimed to identify and characterize children and young adults with premature atherosclerosis at risk for future CVD.

## Methods

### Subjects

The Institutional Review Board of Penn Medicine-Lancaster General Hospital (Lancaster, PA) approved the research study. We screened 570 subjects from our internal DNA biobankfor the *APOB* c.10580G > A variant. We re-contacted individuals/families found to harbor the variant to invite them to participate in the study. Subjects from five families consented to research for themselves or on behalf of their affected children. All subjects were naive to lipid-lowering and antihypertensive medications.

### Genetic testing and serum analysis

To genotype individuals for the *APOB* c.10580G > A variant, we developed a high-resolution melt analysis using an unlabeled probe on a LightScanner 32 System (BioFire Diagnostics, Salt Lake City, UT). We designed the primers (F-TATGCGTTGGAGTGTGGCTTCTCC, R-TGTCAAGGGTTCGGTTCTTTCTCG) and probe (P-CACTGAAGACCGTGTGCTCTTGGAATT) using Primer3 (http://bioinfo.ut.ee/primer3-0.4.0/). We validated the assay in patients, their parents, and siblings of known genotype to demonstrate accurate allele discrimination and genotype calls. Whole exome sequencing was performed by Regeneron Research Center (Tarrytown, NY) as previously described [9]. Lipid analysis and other serum measurements were analyzed from fasting blood by a CLIA-certified laboratory (Health Diagnostic Laboratory, Inc, Richmond, VA).

### Echocardiogram

Baseline echocardiograms were performed using the GE Vivid i imaging system and included all standard views to obtain baseline information and rule out structural defects and aortic stenosis.

### Carotid intima media thickness

All examinations were performed at the start of the day, prior to activity and meals. Height, weight, and resting blood pressure were obtained. A vascular examination was performed utilizing a linear transducer on the GE Vivid i. ECG-gated longitudinal images of the common carotid artery were obtained for both the left and right carotid with focus on capturing the best images of the intima lining proximal to the carotid bulb. CIMT measurements were obtained offline on GE EchoPAC semi-automated software. Triplicate measurements were made on the posterior wall of the vessel 10 mm away from the carotid bulb and averaged. A Meyer’s arc device was used to record the angle of acquisition for reproducibility. All imaging was conducted by a single echo technician and interpreted by one pediatric cardiologist. Both were blinded to the patient’s genotype at the time of data acquisition.

### Pulse wave velocity

Pulse wave velocity was calculated as the traversed distance divided by transit time. Three tracings were obtained per subject and averaged. ECG-gated Doppler image was acquired from the right carotid artery and right femoral artery using the linear probe of the GE Vivid i. After the Doppler images were obtained, the distance (cm) from the site of acquisition to the suprasternal notch was measured to determine the overall distance from the carotid to the femoral artery and determine the site for reproducibility. The ECG gated images were analyzed offline using the GE EchoPAC software to determine transit time. All imaging was conducted by a single echo technician and interpreted by one pediatric cardiologist. Both were blinded to the patient’s genotype at the time of data acquisition.

### Peripheral artery tonometry

Peripheral artery tonometry was measured following an overnight fast and prior to physical activity in the left upper extremity using methods previously described (EndoPAT, Intamar Medical Inc, Caesarea, Israel) [10]. Baseline, occlusion (40–60 mmHg above baseline systolic pressure), and post-occlusion intervals were each 5 min.

### Statistical analysis

ApoB^R3500Q^ heterozygotes were compared to age-matched wild type sibling controls. ApoB^R3500Q^ homozygotes were not included in statistical analysis due to small sample size but are included in tables and figures for completeness. Descriptive summaries with medians (min–max) or percentages are reported due to small sample sizes and the distributions of the data. For comparisons of median values between groups, we utilized a non-parametric test and report p-values using the Wilcoxon rank-sum test. The Fisher’s exact test was used for proportions. We used the non-parametric Spearman method in cases where correlations are reported and added a Bonferroni correction in cases where multiple correlations were calculated. In cases where the Bonferroni correction was used (Fig. 2 = 10 tests and Fig. 3 = 6 tests), we report the corrected p-value of the test calculated *p*-value multiplied by the number of tests so that all reported p-values (familywise or individual) can be assessed relative to our desired alpha. We assessed CIMT, PWV, and reactive hyperemia index (RHI) between the control and heterozygous groups using the Wilxoxon rank-sum test as well as the correlation with age using the Spearman method. In all cases, *p*-values < 0.05 were considered significant and analyses were conducted using Stata version 16.1 (College Station, TX).

## Results

### Cohort demographics

Twenty-five subjects [13 ApoB^R3500Q^ heterozygotes, 3 ApoB^R3500Q^ homozygotes, and 9 age-matched sibling controls, ages 3–28, 60% male] were included in the study. Families were identified by genetic screening for ApoB^R3500Q^ among patients from the Clinic for Special Children, so index cases had medical comorbidities, including limb-girdle muscular dystrophy (n = 2), congenital CMV (n = 1), familial hypercholanemia (n = 1), Trisomy 21 (n = 1), Rett syndrome (n = 1), propionic acidemia (n = 1), and traumatic brain injury (n = 1). No ApoB^R3500Q^ heterozygotes or homozygotes had xanthomas. Whole exome sequence analysis revealed no additional pathogenic variants in *LDLR*, *APOB*, or *PCSK9* that could alter lipid homeostasis or atherosclerotic disease progression. Four control subjects were heterozygous for a pathogenic variant in *ABCG8* (NM_022437.3; c.1720G > A; p.Gly574Arg), indicating carrier status for recessively inherited sitosterolemia.

There was no significant difference in average age, BMI, systolic blood pressure, free fatty acids, or markers of overall metabolic and organ function (hemoglobin A1c, homocysteine, alanine aminotransferase, estimated glomerular filtration rate, thyroid stimulating hormone) between ApoB^R3500Q^ heterozygotes and sibling controls (Table 1). Inflammatory markers (high sensitivity CRP, fibrinogen, lipoprotein-associated phospholipase A2, and myeloperoxidase) were not significantly different in ApoB^R3500Q^ heterozygotes.

**Table 1:** Cohort demographics

	Reference range	Controls (n = 9)	ApoB^R3500Q^		*P*-value
			Heterozygotes (n = 13)	Homozygotes (n = 3)	
Age (years)	NA	16 (7–26)	14 (3–28)	9 (4–22)	0.707
Males [n (%)]	NA	5 (56)	8 (62)	2 (67)	1.000
Body mass index (kg/m^2^)	18.5—24.9	21.5 (15.8–25.6)	17.9 (14.7–24.5)	13.9 (13.8–15.6)	0.124
Systolic blood pressure (mmHg)	NA	110 (96–130)	100 (90–140)	90 (90–120)	0.416
Hemoglobin A1c (%)	≤ 5.6	5.4 (5.1–5.8)	5.2 (4.7–5.7)	5.3 (5.3–5.4)	0.220
Free fatty acids (mmol/L)	< 0.60	0.4 (0.3–0.7)	0.5 (0.2–1.4)	0.7 (0.5–0.7)	0.383
Homocysteine (μmol/L)	< 11	6 (5–12)	6 (4–10)	6 (5–6)	0.853
Alanine aminotransferase (U/L)	< 34	16 (11–87)	18 (8–77)	16 (14–20)	0.831
Estimated GFR (mL/min/1.73 m^2^)	> 89	127 (93–150)	130 (104–150)	91 (79–150)	0.831
Thyroid stimulating hormone (μIU/mL)	0.27–4.20	2.5 (1.6–3.2)	2.5 (0.7–5.6)	3.0 (1.3–3.2)	0.695
High-sensitivity CRP (mg/L)	< 1.0	0.7 (0.3–2.4)	0.3 (0.3–1.9)	1.1 (0.3–11.9)	0.159
Fibrinogen (mg/dL)	126–437	352 (274–513)	333 (239–476)	387 (234–601)	0.647
Lipoprotein-associated phospholipase A2 (ng/mL)	< 200	151 (130–250)	184 (144–239)	226 (161–231)	0.299
Myeloperoxidase (pmol/L)	≤ 320	318 (240–414)	291(207–672)	292 (205–381)	0.209
Total cholesterol (mg/dL)	NA	168 (126–255)	225 (149–297)	396 (333–399)	0.021
LDL-C (mg/dL)	NA	95 (59–154)	165 (101–219)	304 (288–341)	0.001
HDL-C (mg/dL)	NA	71 (45–95)	58 (40–100)	66 (54–87)	0.403
Triglycerides (mg/dL)	NA	32 (28–96)	38 (28–98)	53 (43–56)	0.566

Biometric and clinical data for ApoB^R3500Q^ heterozygotes and homozygotes and age-matched sibling controls in the study cohort. ApoB^R3500Q^ homozygotes were not included in statistical analysis due to small sample size but are presented for completeness. Median (min–max) compared by the exact Wilcoxon rank-sum test unless noted. Fisher’s exact test used where n (%) reported

GFR, Glomerular filtration rate; CRP, C-reactive protein; NA, Not applicable

### Serum lipid analysis

**Fig. 1:**
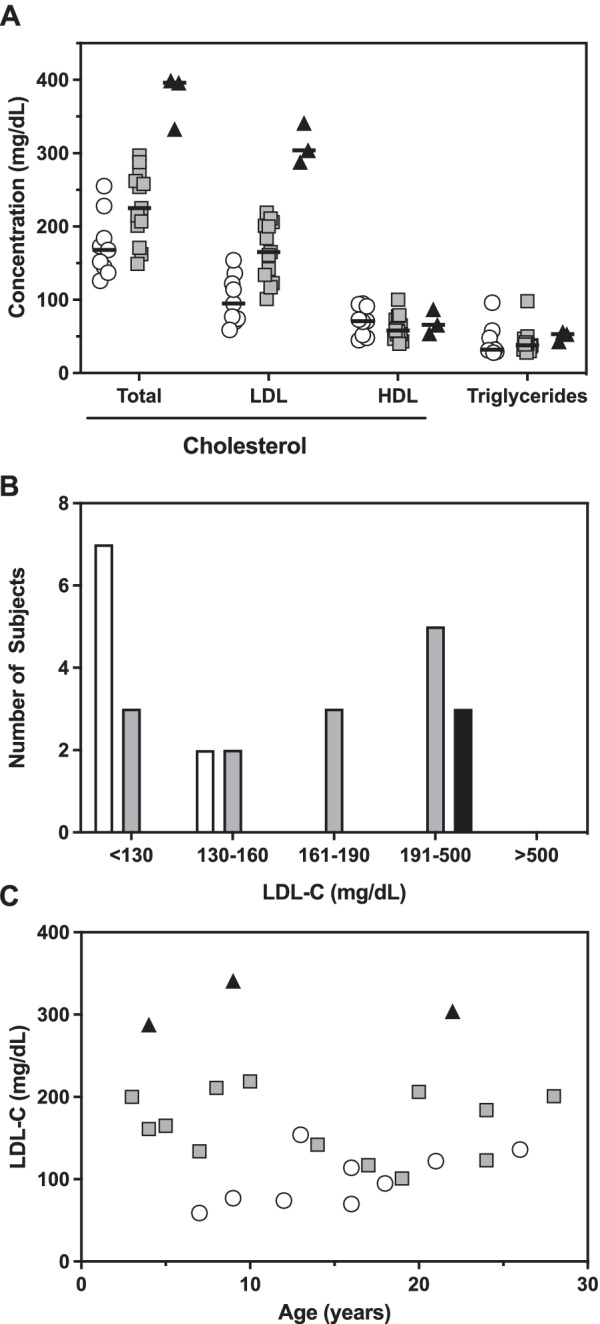
Fasting serum lipid levels. **A** Cholesterol and triglyceride levels for controls (white circles), ApoB^R3500Q^ heterozygotes (gray squares), and ApoB^R3500Q^ homozygotes (black triangles). **B** Several ApoB^R3500Q^ heterozygotes (gray bars) had LDL-C in the normal range (< 130 mg/dL) or below the threshold for suspecting heterozygous FH (160 mg/dL with a family history or 190 mg/dL). All ApoB^R3500Q^ homozygotes (black bar) had LDL-C below 500 mg/dL, the level typically associated with homozygous FH. **C** LDL-C among controls, ApoB^R3500Q^ heterozygotes, and ApoB^R3500Q^ homozygotes (black triangles) compared to subject age. ApoB^R3500Q^ homozygotes (black triangles) were not included in statistical analysis due to small sample size but are presented for completeness. LDL-C did not correlate significantly with age in controls or ApoB^R3500Q^ heterozygotes. LDL-C association with age measured by Spearman’s rho (r_s_)

LDL cholesterol was significantly higher in ApoB^R3500Q^ heterozygotes (median 165, range 101–219 mg/dL) compared to controls (median 95, range 59–154 mg/dL, *P* = 0.001), Table 1 and Fig. 1A. This was reflected in a quantitatively similar increase in total cholesterol in ApoB^R3500Q^ heterozygotes (median 225, range 149–297 mg/dL) compared to controls (median 168, range 126–255 mg/dL, *P* = 0.021). HDL cholesterol and triglycerides were not different among genotypes.

Heterozygous FH is typically suspected in children with LDL-C above 160 mg/dL and a positive family history or LDL-C above 190 mg/dL [6]. In this study of 13 ApoB^R3500Q^ heterozygotes, 3 (23%) had LDL-C above 160 mg/dL and 5 (38%) had LDL above 190 mg/dL (Fig. 1B). All ApoB^R3500Q^ homozygotes had LDL-C above 190 mg/dL, but none above 500 mg/dL, the level typically associated with homozygous FH [1]. LDL-C did not correlate significantly with age in controls or ApoB^R3500Q^ heterozygotes (Fig. 1C).

ApoB^R3500Q^ heterozygotes had significantly higher ApoB (*P* = 0.001), ApoB:ApoA-1 (*P* < 0.001), LDL-P (*P* = 0.001), and sdLDL-P (*P* = 0.003) compared to controls (Table 2). Small, dense LDL cholesterol (as a percent of LDL-C), Lipoprotein(a) [Lp(a)-P], ApoA-1, HDL-P, and HDL2-C were unaffected by genotype.

**Table 2:** Advanced lipid analysis

	Reference Range	Controls (n = 9)	ApoB^R3500Q^		*P*-value
			Heterozygotes (n = 13)	Homozygotes (n = 3)	
ApoB (mg/dL)	< 60	65 (42–103)	117 (77–147)	246 (179–250)	0.001
ApoB:ApoA-1	≤ 0.60	0.5 (0.3–0.8)	0.8 (0.6–1.2)	1.7 (1.5–1.9)	< 0.001
LDL-P (nmol/L)	< 1020	1085 (597–1578)	1818 (1216–2369)	NA	0.001
sdLDL-P (mg/dL)	< 21	17 (10–28)	32 (16–46)	47 (33–62)	0.003
sdLDL-C (% of LDL)	< 26	18 (16–22)	17 (15–24)	16 (10–20)	0.375
Lp(a)-P (nmol/L), n (%) ≥ 75	< 75	4 (44)	4 (31)	NA	0.662
ApoA-1 (mg/dL)	> 150	143 (119–170)	137 (107–168)	135 (119–143)	0.214
HDL-P (umol/L)	> 38	32.8 (31.0–39.8)	35.1 (29.1–42.8)	NA	0.987
HDL2-C (mg/dL)	≥ 17	35 (14–50)	24 (10–52)	19 (14–38)	0.269

Fasting apolipoproteins and small particle analysis for ApoB^R3500Q^ heterozygotes and homozygotes and age-matched sibling controls. *P*-values pertain to comparisons of controls and ApoB^R3500Q^ heterozygotes. ApoB^R3500Q^ homozygotes are included for completeness but not included in statistical analysis. Median (min–max) compared by the exact Wilcoxon rank-sum test unless noted. Fisher’s exact test used where n (%) reported

ApoB, Apolipoprotein B; ApoA-1, Apolipoprotein A-1; LDL-P, Low-density lipoprotein particles; sdLDL-C, Small, dense low density lipoprotein cholesterol; Lp(a)-P, Lipoprotein(a) particles; HDL-P, High density lipoprotein particles; HDL2-C, High-density lipoprotein 2 cholesterol; NA, Insufficient data to report

**Fig. 2:**
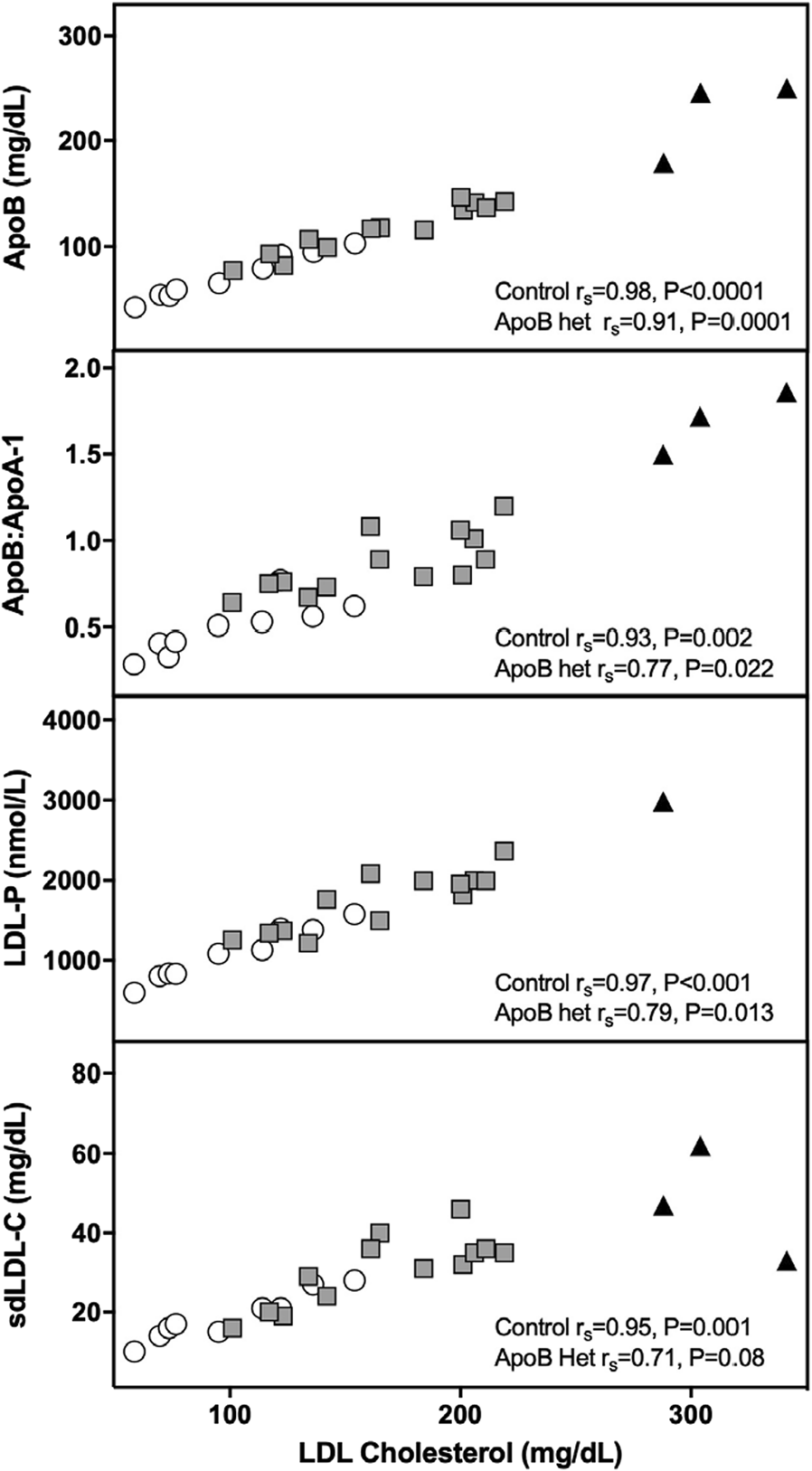
Correlations between LDL-C and lipoproteins and lipid particles. LDL-C correlated significantly with Apolipoprotein B (ApoB), ApoB:ApoA and LDL particles (LDL-P) in controls (white circles) and ApoB^R3500Q^ heterozygotes (gray squares). LDL-C correlated significantly with small dense LDL (sdLDL) in controls, but not ApoB^R3500Q^ heterozygotes. ApoB^R3500Q^ homozygotes (black triangles) were not included in statistical analysis due to small sample size but are presented for completeness. LDL-C association with individual small lipid particles measured by Spearman’s rho (r_s_)

Several apolipoproteins, including ApoB, ApoB:ApoA-1, and LDL-P correlated strongly with LDL-C in both controls and ApoB^R3500Q^ heterozygotes (Fig. 2). Small, dense LDL-C correlated with LDL-C in controls, but not ApoB^R3500Q^ heterozygotes. Additional analysis of plant sterols and fatty acids found nominally significantly higher docosahexaenoic acid (DHA, *P* = 0.034) among ApoB^R3500Q^ heterozygotes compared to controls. No significant differences were noted for campesterol, sitosterol, cholestanol, desmosterol, or omega-3, omega-6, cis-monounsaturated, saturated, or trans fatty acids. (Additional file 1: Supplemental Table 1).

### Cardiovascular imaging

In age-adjusted analysis, CIMT, PWV, and RHI were not significantly different between ApoB^R3500Q^ heterozygotes and controls. No subjects (controls, ApoB^R3500Q^ heterozygotes or homozygotes) had supravalvular aortic stenosis or structural cardiac defects.

**Fig. 3:**
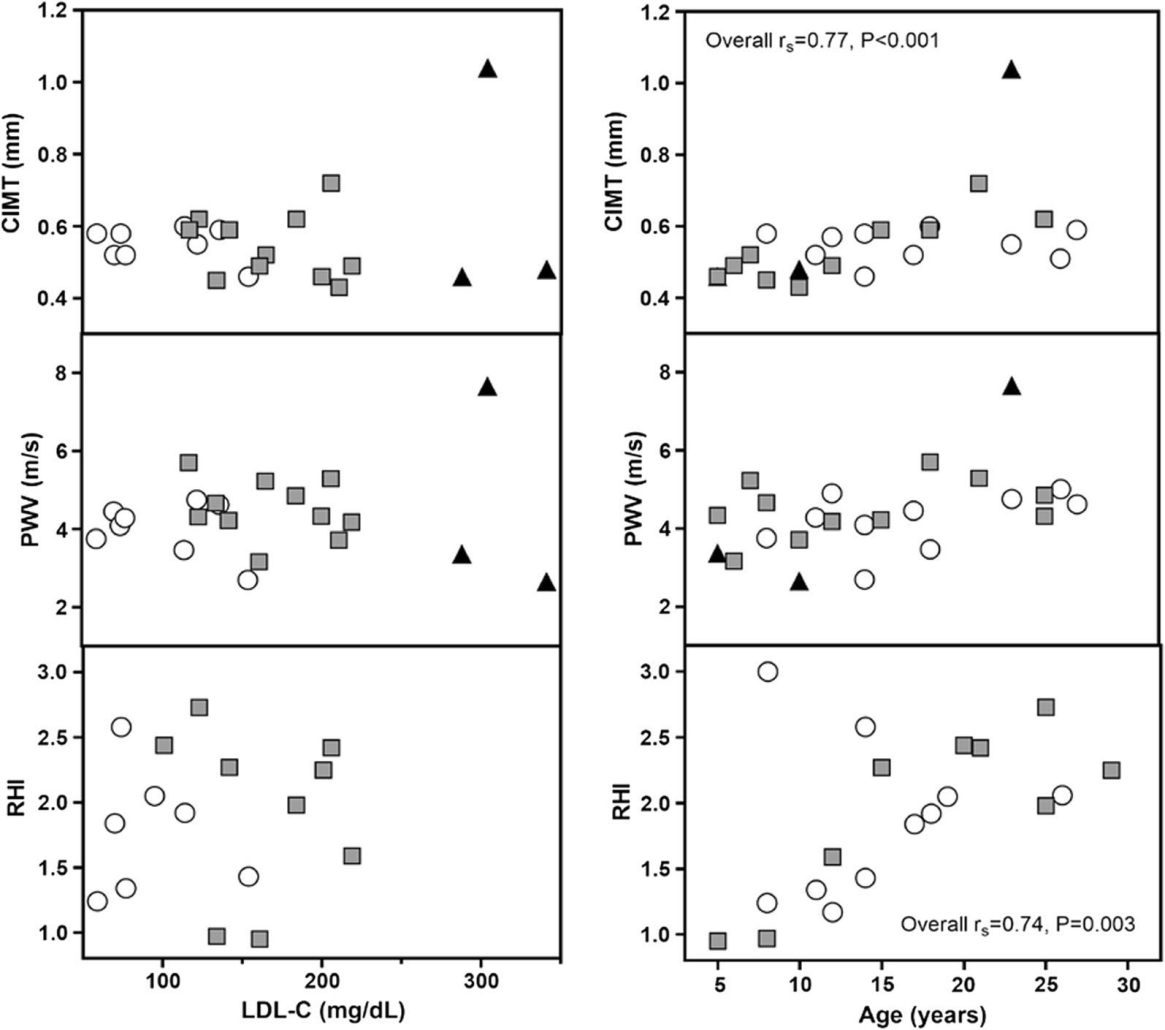
Endothelial function, vascular stiffness, and atherosclerotic plaque. Carotid intima media thickness (CIMT, a surrogate for atherosclerotic plaque burden), pulse wave velocity (PWV, a measure of vascular stiffness), and reactive hyperemia index (RHI, a measure of endothelial function) were similar in ApoB^R3500Q^ heterozygotes (gray squares) and controls (white circles) and did not correlate with LDL-C or age in either group. CIMT and RHI correlated with age in overall subjects. ApoB^R3500Q^ homozygotes (black triangles) were not included in statistical analysis due to small sample size but are presented for completeness. Imaging values association with LDL-C or age measured by Spearman’s rho (r_s_)

CIMT is a measure of atherosclerotic plaque and previous studies report CIMT between 0.38–0.5 mm in healthy children and young adults [11]. Our CIMT measurements were within this range for 10% of controls, 45% of ApoB^R3500Q^ heterozygotes, and 66% of ApoB^R3500Q^ homozygotes, likely reflecting variation in operator-dependent aspects of technique. CIMT measurements did not correlate with LDL-C. There was a correlation between CIMT and age when considering all study subjects in aggregate (overall r_s_ = 0.77, *P* < 0.001) but no such correlations were found among genotype subgroups (controls or ApoB^R3500Q^ heterozygotes, Fig. 3, top panels). Of note, the interaction effect of LDL-C and age was also significant (coefficient = 0.0001, *P* = 0.025) in predicting CIMT. We found no significant correlation between CIMT and sdLDL or LDL-P (data not shown).

Normal values for PWV are not firmly established, but previous studies report values between 4.1 and 10.9 m/s for control subjects [11]. Our PWV measurements were within or below this range in all subjects. PWV did not correlate with age or LDL-C in overall subjects, controls or ApoB^R3500Q^ homozygotes (Fig. 3, middle panels). We found no significant correlation between PWV and sdLDL or LDL-P (data not shown).

RHI ≤ 1.67 reflects abnormal nitric oxide-dependent changes in vascular tone and presumably early evidence of CVD. In the current cohort, RHI values were below the previously described normal range (≤ 1.67) for the majority (n = 9, 75%) of subjects under age 20, regardless of genotype (Fig. 3, bottom panels). RHI did not correlate with LDL-C but correlated with age in overall subjects (overall r_s_ = 0.74, *P* = 0.003), but not subgroups (controls or ApoB^R3500Q^ heterozygotes). Of note, RHI values could not be obtained from 4 subjects (1 control, 1 ApoB^R3500Q^ heterozygote, and 2 ApoB^R3500Q^ homozygotes) due to disability or inability to be still for examination. Five additional values (1 control, 3 ApoB^R3500Q^ heterozygotes, and 1 ApoB^R3500Q^ homozygote) were excluded from the analysis due to poor data quality due to improper probe fitting on small fingers in young subjects. We found no significant correlation between RHI and sdLDL or LDL-P (data not shown).

## Discussion

Here, we report advanced lipid testing, cardiovascular imaging, vascular stiffness, and endothelial function for a cohort of children and young adults with the Amish ApoB^R3500Q^ founder variant. Previous studies in children with FH, predominantly LDL receptor defects, have demonstrated higher CIMT among affected children compared to unaffected siblings beginning at 8 to 12 years of age [12]. We found lower LDL-C levels than seen with other forms of FH, increased number of LDL particles, and absence of detectable atherosclerosis.

### Advanced lipid testing

Apolipoprotein B^R3500Q^ increases circulating LDL-C and coronary calcification in adults [5]. Comparing LDL-C between adults and children/adolescents with ApoB^R3500Q^ is complicated by the fact that cholesterol levels normally peak around age 9–11 years, fall during adolescence, and increase progressively after age 17 years [13–16]. Collectively, we find children and adolescents with ApoB^R3500Q^ have elevations of LDL-C compared to age-matched controls, similar to what is observed in adults with the same gene variant. However, our study size was not large enough to demonstrate the expected peak of LDL-C at age 9–11 years and after 17 years. Interestingly, several subjects with ApoB^R3500Q^ had normal or only slightly elevated LDL-C and would thus be overlooked using current cholesterol screening guidelines [17]. This is consistent with previous reports of incomplete penetrance for ApoB variants [18] and underscores the importance of cascade genotyping (rather than lipid screening alone) to identify FH in at-risk family members. The variability in LDL-C elevations among ApoB^R3500Q^ heterozygotes may also be influenced by diet or other environmental factors.

In addition to LDL-C, other serum lipids are associated with atherosclerosis risk in adults, but their predictive utility in pediatrics is largely unknown. All pro-atherogenic lipoproteins, including LDL-C, contain one ApoB surface protein, so serum measurements of ApoB or the ratio of ApoB to ApoA-1 (the primary lipoprotein on HDL, a protective lipoprotein) may more accurately reflect the number of atherogenic particles and better predict CVD risk. Some studies support this hypothesis [19–24], while others have found ApoB and ApoB:ApoA-1 to be equivocal to LDL-C in predicting CVD risk [25–28]. Consistent with this observation, ApoB^R3500Q^ heterozygotes had elevations of ApoB and ApoB:ApoA-1 that were strongly correlated with LDL-C, suggesting that in patients with FH, these biomarkers might be interchangeable with predicted CVD risk.

Cholesterol content in LDL is variable; some particles are large and cholesterol-rich, whereas others are small and dense. LDL particle number (LDL-P) has been shown to be a stronger predictor of CVD risk compared to LDL-C in some studies, particularly when there is discordance between LDL-C and LDL-P [29–31], while others have shown its predictive value to be comparable to LDL-C [32]. ApoB^R3500Q^ heterozygotes had significantly increased LDL-P and sdLDL-C and LDL-P correlated strongly with LDL-C.

Lipoprotein(a) consists of a single ApoB with a plasminogen-like protein [apoprotein(a)]. Lipoprotein(a) levels vary greatly among individuals and do not correlate with LDL-C, non-HDL-C, ApoB, or LDL particle number [33]. Lp(a) has been shown to be predictive of CVD risk in adults, independent of LDL-C, especially in those with elevated LDL-C and FH [34–37]*.* Lp(a) is higher in individuals with FH and it is postulated that severe elevations of Lp(a) have an FH-like clinical phenotype [38–40]. In our study, Lp(a) concentrations were similar among controls and ApoB^R3500Q^ heterozygotes.

Our study also noted significantly increased docosahexaenoic acid (DHA) among ApoB^R3500Q^ heterozygotes compared to controls. Previous studies among Amish adults demonstrated increased sitosterol, campesterol, and stigmasterol associated with heterozygosity for a variant in *ABCG8* [41]. This is the first report, to our knowledge, of baseline differences in circulating levels of DHA in FH. Subjects were not asked to report dietary supplement use, so this may be an artifact of supplement use or a true increase in circulating DHA among those with a particular genotype.

### Cardiovascular function and anatomy

Contrary to previous reports of FH in children, we did not find increases in CIMT among ApoB^R3500Q^ heterozygotes [42]. In overall subjects, CIMT significantly correlated with age, but this relationship was not significant in controls or ApoB^R3500Q^ heterozygote subgroups. The interaction effect of LDL-C and age was significant, suggesting a cumulative effect of age and LDL-C on CIMT. However, given the small cohort in our study it is difficult to draw a definitive conclusion from this interaction. The study cohort had lower LDL-C values than seen in other forms of FH, which may explain the lack of differences in CIMT.

Pulse wave velocity is a measure of vascular stiffness and can be measured by a variety of methods, including ultrasound and cardiac MRI. Studies using a variety of testing methods show PWV in healthy pediatric subjects to be quite variable [11] and increased with age [43]. Here, we measured PWV with ultrasound and detected values similar to previous reports for control subjects. In contrast to increased PWV previously reported in children with FH [44], PWV among ApoB^R3500Q^ heterozygotes was indistinguishable from controls and we found no significant correlations between PWV and either age or LDL-C.

Abnormal flow-mediated dilation is found in children with a family history of cardiovascular events, FH, and familial combined hyperlipidemia in other studies [45]. We found similar “abnormal” flow mediated dilation in the majority of subjects under age 20 years, regardless of genotype, with improvement to normal ranges after age 20 years. This is contrary to what would be expected based on pathophysiology and raises concern that this imaging technique was inaccurate in our study subjects under age 20 years.

## Strengths and limitations

There are few detailed studies of children with FH, and those that exist consist of subjects with a heterogeneous genetic makeup, most commonly LDL receptor defects. We focused on a cohort with identical ApoB^R3500Q^ variants, similar lifestyle, and relatively homogeneous environmental exposures, and compared them to age-matched, sibling controls to limit variation from additional genetic and environmental factors. To further limit spurious or unforeseen background influences, we excluded any other pathogenic *APOB*, *LDLR*, *PCSK9, or ABCG8* variants by exome sequencing for all study subjects. Several subjects had medical comorbidities unrelated to lipid metabolism that have not been described in previous FH cohorts. Finally, we utilized contemporary imaging techniques, including advanced lipid testing and cardiovascular imaging, which have mainly been studied in adults. The size of our cohort was small and the variant has incomplete penetrance, which may have constrained our ability to detect subtle changes in laboratory values or cardiovascular imaging indices that may have reached statistical significance with a larger sample size. Our study did not include dietary record analysis or supplement use, so relationships between nutrient intake and lipid levels and/or cardiovascular imaging could not be assessed.

## Conclusions

We provide a detailed description of the serum lipids, atherosclerotic plaque burden, vascular stiffness, and endothelial function among children and young adults with FH due to ApoB^R3500Q^. Contrary to previous reports of middle-age and older adults with ApoB^R3500Q^ [2], we did not find any difference in atherosclerotic measures in children and young adults with ApoB^R3500Q^ compared to controls. Fasting LDL-C was higher in children with ApoB^R3500Q^ compared to controls, but normal or below the threshold for suspecting FH for several ApoB^R3500Q^ heterozygotes, emphasizing the importance of cascade genotyping (rather than fasting cholesterol levels alone) among related individuals for diagnosis and early treatment.

## Supplementary Information

**Additional file 1**. Supplemental Table 1: Plant sterols and fatty acids. Fasting plant sterol and fatty acid levels for ApoB^R3500Q^ heterozygotes, homozygotes and age-matched sibling controls. Median (min-max) compared by the exact Wilcoxon rank-sum test.

## Data Availability

The datasets used and/or analysed during the current study are available from the corresponding author upon reasonable request.

## Abbreviations

FH: Familial hypercholesterolemia
CIMT: Carotid intima media thickness
PWV: Pulse wave velocity
LDL-C: LDL cholesterol
CVD: Cardiovascular disease
ApoB: Apolipoprotein-B
ApoA-1: Apolipoprotein A-1
LDL-P: LDL particles
HDL-P: HDL particles
sdLDL-C: Small, dense LDL cholesterol
Lp(a): Lipoprotein(a)
RHI: Reactive hyperemia index
