# Supplementary material for: Clinical characterization of familial hypercholesterolemia due to an amish founder mutation in Apolipoprotein B
Source: BMC Cardiovasc Disord. 2022 Mar 17;22:109. doi: 10.1186/s12872-022-02539-3 (PMC8928591; doi:10.1186/s12872-022-02539-3)
Supplement: Supplementary file 1 — Additional file 1. Supplemental Table 1: Plant sterols and fatty acids. Fasting plant sterol and fatty acid levels for ApoBR3500Q heterozygotes, homozygotes and age-matched sibling controls. Median (min-max) compared by the exact Wilcoxon rank-sum test. [file 12872_2022_2539_MOESM1_ESM.docx]

**Supplemental Table 1:** **Plant sterols and fatty acids.** Fasting plant sterol and fatty acid levels for ApoB^R3500Q^ heterozygotes, homozygotes and age-matched sibling controls. Median (min-max) compared by the exact Wilcoxon rank-sum test.

|  |  |  |  | **ApoB^R3500Q^** | |  |
| --- | --- | --- | --- | --- | --- | --- |
|  |  | **Reference Range** | **Controls**  (n=9) | **Heterozygotes**  (n=16) | **Homozygotes**  (n=3) | **P-value** |
| **Plant Sterols** | **Campesterol** (μg/mL) | 2.11 - 4.43 | 3.0 (1.3-6.3) | 4.3 (1.5-7.8) | 7.0 (5.1-11.1) | 0.209 |
|  | **Campesterol Ratio** (100 mmol/mol cholesterol) | 115 - 240 | 149 (86-265) | 142 (73-365) | 171 (148-268) | 0.896 |
|  | **Sitosterol** (μg/mL) | 1.43 - 3.17 | 1.9 (0.9-3.6) | 3.0 (1.2-4.8) | 3.7 (3.1-7.3) | 0.164 |
|  | **Sitosterol Ratio** (100 mmol/mol cholesterol) | 76 - 168 | 104 (57-207) | 99 (57-200) | 100.5 (72-170) | 0.961 |
|  | **Cholestanol** (μg/mL) | 2.02 - 3.47 | 2.7 (1.5-4.1) | 3.5 (1.9-4.6) | 5.5 (3.9-7.8) | 0.140 |
|  | **Cholestanol Ratio** (100 mmol/mol cholesterol) | 117 - 194 | 144 (110-215) | 142 (101-194) | 136 (116-196) | 0.896 |
|  | **Desmosterol** (μg/mL) | 0.50 - 1.27 | 1.1 (1.0-1.5) | 1.4 (1.0-4.9) | 2.1 (1.0-2.4) | 0.099 |
|  | **Desmosterol Ratio** (100 mmol/mol cholesterol) | 31 - 64 | 71 (49-82) | 61 (49-166) | 53 (26-72) | 0.383 |
| **Fatty Acids** | **Omega-3 Total** (%) | 0.1 - 14.1 | 7.3 (6.6-7.6) | 7.5 (6.4-9.7) | 7.8 (7.1-7.9) | 0.156 |
|  | **Alpha-linoleic (ALA)** (%) | 0.1 - 0.4 | 0.1 (0.1-0.2) | 0.1 (0.1-0.2) | 0.1 (0.1-0.2) | 1.000 |
|  | **Docosapentaenoic (DPA)** (%) | 0.6 - 4.1 | 2.8 (2.1-3.2) | 2.5 (2.2-3.1) | 2.5 (2.3-3.6) | 0.801 |
|  | **Eicosapentaenoic (EPA)** (%) | 0.1 - 2.5 | 0.4 (0.3-0.5) | 0.4 (0.3-0.8) | 0.5 (0.5-0.6) | 1.000 |
|  | **Docosahexaenoic (DHA)** (%) | 0.1 - 8.4 | 4.0 (3.8-4.2) | 4.3 (3.3-6.7) | 4.7 (2.6-4.9) | 0.034 |
|  | **Omega-6 Total** (%) | 28.6 - 44.5 | 38.3 (37.6-38.7) | 37.6 (33.9-39.9) | 36.9 (35.9-37) | 0.085 |
|  | **Arachidonic (AA)** (%) | 10.5 - 23.3 | 17.9 (17-18.5) | 17.5 (15.5-18.4) | 17.1 (17.0-17.2) | 0.180 |
|  | **Linoleic (LA)** (%) | 4.6 - 21.3 | 12.8 (11.9-13.7) | 12.1 (10.9-15.3) | 12.7 (11.9-13.2) | 0.331 |
|  | **cis-Monounsaturated Total** (%) | 11.5 - 20.5 | 13.7 (12.5-14.4) | 13.8 (12.6-15.6) | 14.4 (14.2-16.9) | 0.480 |
|  | **Saturated Total** (%) | 36.6 - 42.0 | 40.2 (39.0-40.8) | 40.1 (39.4-41.3) | 40.0 (39.7-40.0) | 0.987 |
|  | **Trans Total** (%) | < 0.1 - 1.8 | 1.0 (0.9-1.2) | 1.0 (0.8-1.2) | 0.9 (0.3-1.0) | 0.564 |
